# Impact of prematurity on long-stay paediatric intensive care unit admissions in England 2008-2018

**DOI:** 10.1186/s12887-023-04254-0

**Published:** 2023-08-24

**Authors:** Tim J. van Hasselt, Hari Krishnan Kanthimathinathan, Trishul Kothari, Adrian Plunkett, Chris Gale, Elizabeth S. Draper, Sarah E. Seaton

**Affiliations:** 1 Department of Population Health Sciences, University of Leicester, Leicester, LE1 7RH UK University Rd,; 2 Paediatric Intensive Care Unit, Birmingham Women’s and Children’s NHS Foundation Trust, Birmingham, UK; 3 Birmingham Women’s and Children’s NHS Foundation Trust, Birmingham, UK; 4 School of Public Health, Faculty of Medicine, Neonatal Medicine, Imperial College London, London, UK

**Keywords:** Pediatric intensive care units, Prematurity, Neonatal, Neonatal intensive care, Chronic disease

## Abstract

**Background:**

Survival following extreme preterm birth has improved, potentially increasing the number of children with ongoing morbidity requiring intensive care in childhood. Previous single-centre studies have suggested that long-stay admissions in paediatric intensive care units (PICUs) are increasing.

We aimed to examine trends in long-stay admissions (≥28 days) to PICUs in England, outcomes for this group (including mortality and PICU readmission), and to determine the contribution of preterm-born children to the long-stay population, in children aged <2 years.

**Methods:**

Data was obtained from the Paediatric Intensive Care Audit Network (PICANet) for all children <2 years admitted to National Health Service PICUs from 1/1/2008 to 31/12/2018 in England. We performed descriptive analysis of child characteristics and PICU outcomes.

**Results:**

There were 99,057 admissions from 67,615 children. 2,693 children (4.0%) had 3,127 long-stays. Between 2008 and 2018 the annual number of long-stay admissions increased from 225 (2.7%) to 355 (4.0%), and the proportion of bed days in PICUs occupied by long-stay admissions increased from 24.2% to 33.2%.

Of children with long-stays, 33.5% were born preterm, 53.5% were born at term, and 13.1% had missing data for gestational age.

A considerable proportion of long-stay children required PICU readmission before two years of age (76.3% for preterm-born children). Observed mortality during any admission was also disproportionately greater for long-stay children (26.5% for term-born, 24.8% for preterm-born) than the overall rate (6.3%).

**Conclusions:**

Long-stays accounted for an increasing proportion of PICU activity in England between 2008 and 2018. Children born preterm were over-represented in the long-stay population compared to the national preterm birth rate (8%).

These results have significant implications for future research into paediatric morbidity, and for planning future PICU service provision.

**Supplementary Information:**

The online version contains supplementary material available at 10.1186/s12887-023-04254-0.

## Background

Capacity in paediatric intensive care units (PICUs) is often limited, while demand is increasing [1, 2]. While the median length of stay for the majority of children who require a PICU admission is around two days, a minority of children stay for much longer [3]. Previous research from a range of high-income countries has shown that long-stay patients in PICUs (commonly defined as admitted for 28 days or more [4]) are increasing as a proportion of total admissions, and may account for almost half of PICU bed capacity [4, 5]. This trend is occurring against the background of a general increase in demand on PICUs over time, independent of population growth or birth rate [1]. Children with long-stays have a higher mortality rate compared with that of the general PICU population [3, 4].

At the same time, advances in maternal and neonatal care have led to increased survival rates for babies born preterm, particularly at extremely low gestations [6]. Preterm birth is associated with increased prevalence of chronic disease in survivors, particularly in those born extremely preterm (under 28 weeks) [7]. Bronchopulmonary dysplasia (BPD) is the most common morbidity following very preterm birth and is associated with PICU admission [8]. Despite changes in neonatal respiratory care, there is little evidence that the prevalence of this condition is declining, and may be increasing for the most preterm babies [9]. The increasing numbers of survivors of preterm birth, many of whom have ongoing morbidity, is likely to further increase demand on paediatric intensive care services [6] and may result in higher numbers of long-stay PICU patients.

We aimed to examine trends and outcomes in long-stay admissions to PICUs in England, and to determine the contribution of preterm-born children to the long-stay population.

## Methods

### Study population

All children aged under two years admitted to National Health Service (NHS) PICUs from 1/1/2008 to 31/12/2018 in England were included.

### Data sources

Information about the care children received in PICUs was obtained from the Paediatric Intensive Care Audit Network, PICANet [3], which collects demographic and clinical data for every PICU admission, with complete data for England from 2003 onwards. We extracted information related to admission and child characteristics and the care received. Further details of data standards and validation are available from PICANet Annual Reports, and details of data items are available from https://www.picanet.org.uk. PICANet has Research Ethics Committee (MREC) approval (Ref 18/EM/0267) and National Information Governance Board (4-07(c)/2002-PICANet) approval, utilised for this study.

### Definitions

Long-stay was defined as PICU length of stay of 28 days or more [4]. Standard stay was defined as length of stay under 28 days. Any long-stay admission was defined as any long-stay PICU admission up to the age of 2 years between 2008 and 2018.

Gestational age at birth was defined as per World Health Organisation definitions: 22+0 to 27+6 “extreme preterm”; 28+0 to 31+6 “very preterm”; 32+0 to 36+6 “moderate/late preterm”; 37+0 to 42+6 “term”. Admissions with <22 or ≥43 completed weeks of gestation were assigned as missing. Where present, relevant clinical codes (e.g. for “Gestation = 24 weeks”) within primary diagnosis, other diagnosis, and comorbidity variables were used to assign gestational age at birth if missing. For children with multiple admissions, the modal value for gestational age at birth was used if there were missing values or discrepancies between admissions. Postmenstrual age (PMA) at time of PICU admission was calculated from gestational age at birth and the chronological age at admission.

Primary admission diagnosis was assigned by treating clinicians within PICUs and coded within the PICANet dataset. PICANet diagnostic groups were used to categorise these (e.g. respiratory admissions) [3].

Probability of death calculated on admission using the Paediatric Index of Mortality Version 2 (PIM2) was used as a proxy marker of illness severity. PIM Version 2 was chosen as this was consistently used throughout the timeframe of analysis [3].

### Statistical analysis

We performed separate analyses for trends in total admissions, comparisons by individual child, and trends and comparisons by PICU care days. We compared children by gestation at birth (term, preterm, and extreme preterm), and whether they had standard stays or any long-stay admissions. Frequencies and percentages are presented for categorical or binary variables. Continuous variables were non-normally distributed, so presented with median and interquartile range. Comparisons were made between groups using Mann-Whitney and Χ^2^ tests for continuous and categorical or binary variables respectively. The Mann-Kendall test for trend was used to determine significant of trends over time. Lowess curves were applied to the scatter plots of long-stay admissions against year by gestational at birth, to smooth year-to-year variability [10].

We performed logistic regression analysis using the outcome of any long-stay up to the age of two years, using the predictor variables of: gestation at birth (categorised), age at first PICU admission (corrected if born <37 weeks), sex, diagnostic category of first PICU admission, PIM2 score on first PICU admission, and previous neonatal or intensive care during the hospital episode leading to the first PICU admission. We planned sensitivity analysis due to missing data for gestational age at birth, first assuming that missing values were birth at term, and then including only data from PICUs with high data completeness (<20% missing). The c-statistic and Brier score were used to judge predictive ability of models.

Stata 17.0 (StataCorp. College Station TX USA, 2021) was used for all analysis.

## Results

There were 99,071 admissions to PICUs in England from children aged under two years (56.9% of the 174,164 admissions aged 0-18 years) in 2008 to 2018. We analysed 99,057 admissions from 67,615 children after excluding 14 admissions due to missing length of stay data (see Additional file, Supplementary Figure 1 – flow chart). Gestational ages of six children reported as <22 weeks, and 29 children as ≥43 weeks were considered missing prior to further analysis, although they remained in the study population within the missing gestation group.

A total of 2,693 children (4.0%) had 3,127 long-stays in PICU, as some children had repeated long-stay admissions.

### Comparison of children with standard stay and long-stay admissions

Of the 2,693 children with long-stay admissions, 53.5% were born at term, 33.5% were born preterm, and 8.4% were born extremely preterm. 13.1% of long-stay children had missing data for gestational age. We present comparisons of children with long-stay and standard stay, by gestation at birth (Table 1).

**Table 1:** Comparison between children with any PICU admission ≥28 days (long-stay) and <28 days (standard stay) up to 2 years of age

	Study population	Term^**a**^		Preterm^**a**^					
				All preterm			Extreme preterm (<28 weeks)		
		Standard stay	Long-stay	Standard stay	Long-stay	*p* (long- stay: preterm vs term)	Standard stay	Long-stay	*p* (long-stay: extreme preterm vs term)
Children, n (%)	67,615 (100)	34,457 (96.0)	1,440 (4.0)	15,443 (94.5)	901 (5.5)	<0.001	3,339 (93.6)	227 (6.4)	<0.001
Postmenstrual age at first admission, median weeks (IQR)	42.3 (38.0 to 62.7)	45.3 (40.0 to 70.0)	41.3 (38.0 to 54.1)	38.3 (35.0 to 48.4)	38.3 (34.0 to 46.9)	<0.001	37.9 (29.3 to 47.4)	40.1 (31.3 to 48.4)	<0.001
Female sex, n (%)	28,477 (42.1)	14,519 (42.1)	652 (45.3)	6,421 (41.6)	368 (40.8)	0.034	1,396 (41.8)	88 (38.8)	0.065
Cumulative PICU length of stay, median days (IQR)	3.8 (1.7 to 8.0)	3.7 (1.7 to 7.2)	64.4 (43.0 to 104.9)	4.5 (2.0 to 9.1)	63.8 (43.5 to 102.3)	0.635	4.7 (1.7 to 10.8)	65.6 (44.0 to 103.8)	0.816
Cumulative length of invasive ventilation, median days (IQR)	3 (1 to 7)	3 (1 to 6)	46 (30 to 77.5)	4 (1 to 7)	45 (29 to 74)	0.747	4 (1 to 9)	48 (26 to 79)	0.568
Transfer from neonatal unit on first PICU admission, n (%)	12,528 (18.5)	5,649 (16.4)	432 (30.0)	4,723 (30.6)	467 (51.8)	<0.001	1,612 (48.3)	144 (63.4)	<0.001
Diagnostic category of first PICU admission, n (%)									
Cardiovascular	23,455 (34.7)	13,646 (39.6)	742 (51.5)	3,209 (20.8)	265 (29.4)	<0.001	642 (19.2)	37 (16.3)	<0.001
Respiratory	20,394 (30.2)	9,352 (27.1)	345 (24.0)	6,291 (40.7)	336 (37.3)		1,209 (36.2)	118 (52.0)	
Neurological	5,660 (8.4)	3,012 (8.7)	72 (5.0)	904 (5.9)	46 (5.1)		190 (5.7)	12 (5.3)	
Gastrointestinal	5,359 (7.9)	2,251 (6.5)	64 (4.4)	2,009 (13.0)	90 (10.0)		707 (21.2)	33 (14.5)	
Infection	3,954 (5.9)	1,960 (5.7)	44 (3.1)	1,003 (6.5)	39 (4.3)		157 (4.7	10 (4.4)	
Other	8,584 (12.7)	4,157 (12.1)	169 (11.7)	1,982 (12.8)	125 (13.9)		423 (12.7)	17 (7.5)	
* Missing*	*209 (0.3)*	*79 (0.2)*	*4 (0.3)*	*45 (0.3)*	*0*		11 (0.3)	*0*	
PIM2 on first admission, median % (IQR)	2.2 (1.0 to 5.8)	2.3 (1.1 to 5.7)	4.9 (1.8 to 12.5)	2.1 (1.0 to 6.1)	4.2 (1.5 to 9.6)	0.004	2.8 (0.9 to 8.0)	4.5 (1.6 to 11.0)	0.362
Death within PICU									
First admission, n (%)	2,871 (4.3)	1,397 (4.1)	140 (9.7)	663 (4.3)	86 (9.5)	0.888	154 (4.6)	20 (8.8)	0.665
Any admission, n (%)	4,224 (6.3)	1,944 (5.6)	382 (26.5)	948 (6.1)	223 (24.8)	0.339	220 (6.6)	54 (23.8)	0.383
Readmission to PICU (first admissions 2008-2016), n (%)									
>1 admission, n (%)	14,341 (25.8)	7,419 (26.5)	848 (75.7)	3,429 (27.5)	555 (76.3)	0.733	901 (33.3)	153 (80.1)	0.181
Number of admissions, median (IQR)	1 (1 to 2)	1 (1 to 2)	3 (2 to 5)	1 (1 to 2)	3 (2 to 5)	0.677	1 (1 to 2)	3 (2 to 5)	0.823

*LOS* Length of PICU stay, *IQR* Interquartile range, *PICU* Paediatric intensive care unit, *PIM2* Probability of death (%) by Paediatric Index of Mortality 2 score

^a^15,374 children (22.7%) had missing data for gestation at birth, therefore not shown in comparison by gestation

The median PMA at first PICU admission for preterm-born children was 38 weeks, increasing to 40 weeks for extreme-preterm born children with long-stays. For term-born children the median PMA was 45 weeks at first admission for standard stay children, and 41 weeks for the long-stay group. Across all groups the majority of children were male, with similar proportions between groups.

The median cumulative length of stay and median duration of ventilation were higher among children with long-stays, as expected, however these children also had a median of three PICU admissions before the age of two years, whereas for children with standard stays the median number of PICU admissions was one. The proportion of children requiring multiple admissions was highest in the extreme preterm-born children with long-stays (80.1%).

The proportion of children transferred from neonatal care to PICU for their first admission increased for those born preterm, and for those with long-stays, rising from 16.4% for term-born children with standard stays to 63.4% for extreme-preterm born children with long-stays.

Among term-born children, cardiovascular admissions were the most frequent, particularly among long-stay children (51.5% of first admissions), whereas for the extreme preterm-born children the most common reason for admission was respiratory (52.0% of first admissions of long-stay children born <28 weeks).

The observed mortality rate (death in PICU on any admission) was greater among children with long-stays (23.8% to 26.5%) compared to standard stays (5.6% to 6.6%), however there was no increase in mortality among preterm-born children with long-stays compared with those born at term.

### Prediction of long-stay

We performed multivariable logistic regression to examine whether gestation at birth was associated with any long-stay admission, after adjustment for characteristics at first PICU admission: corrected age, sex, primary diagnostic grouping, PIM2, and previous neonatal or intensive care during the same hospitalisation (see Additional file, Supplementary Table 1). Compared to term-born children, those born preterm had a greater adjusted odds ratio (aOR) for long-stay: for moderate/late preterm-born children the aOR was 1.276 (95% confidence interval, 95%CI: 1.142 to 1.426); for those born very preterm the aOR was 1.348 (95%CI: 1.146 to 1.585), and for extreme preterm-born children it was 1.303 (95%CI: 1.116 to 1.521). However, PIM2 greater than 15% (aOR 3.025 compared with PIM2 <1%), previous PICU admission during the hospital episode (aOR 2.705) or transfer from neonatal care (aOR 1.939) had greater association with long-stay.

The model had moderate predictive ability (c-statistic 0.685, Brier score 0.0813). Results were confirmed in sensitivity analyses where missing gestation was assumed to be birth at term, and where only data from units with high completeness was included.

### Trends in long-stay admissions

**Fig. 1:**
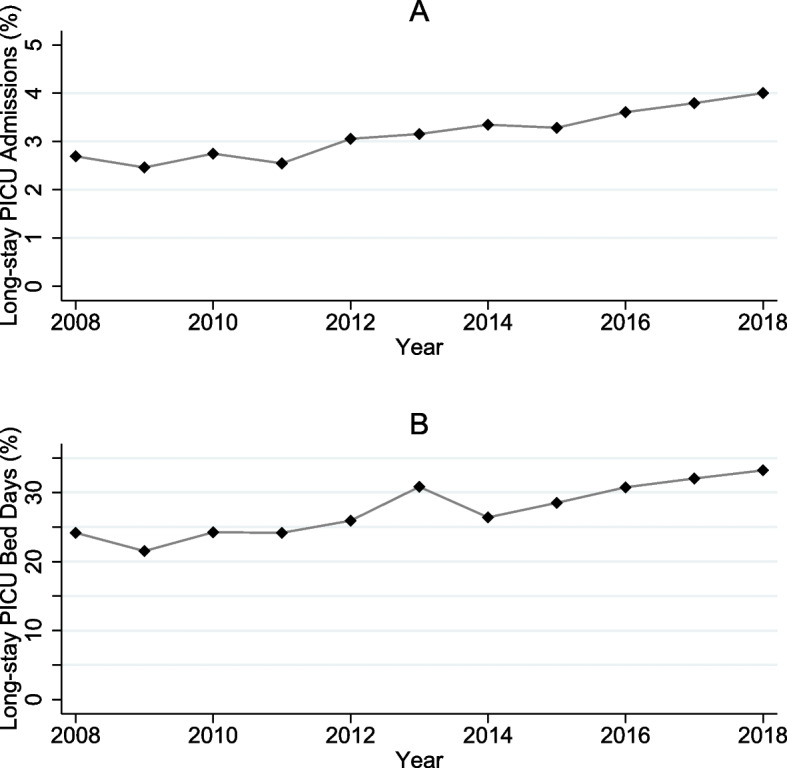
Trends in percentage of long-stay PICU admissions as a proportion of total PICU admissions (**A**) and percentage of long-stay PICU bed days as a proportion of total bed days (**B**), children admitted to PICU aged <2 years in England 2008-2018

There was a clear increase in the percentage of long-stay admissions (Fig 1A), from 2.7% of all admissions in 2008 to 4.0% in 2018. Similarly, the proportion of bed days in PICUs occupied by long-stay admissions increased over the period from under a quarter (24.2% in 2008) to almost one third (33.2% in 2018) of annual bed days over the period (Fig 1B).

**Fig. 2:**
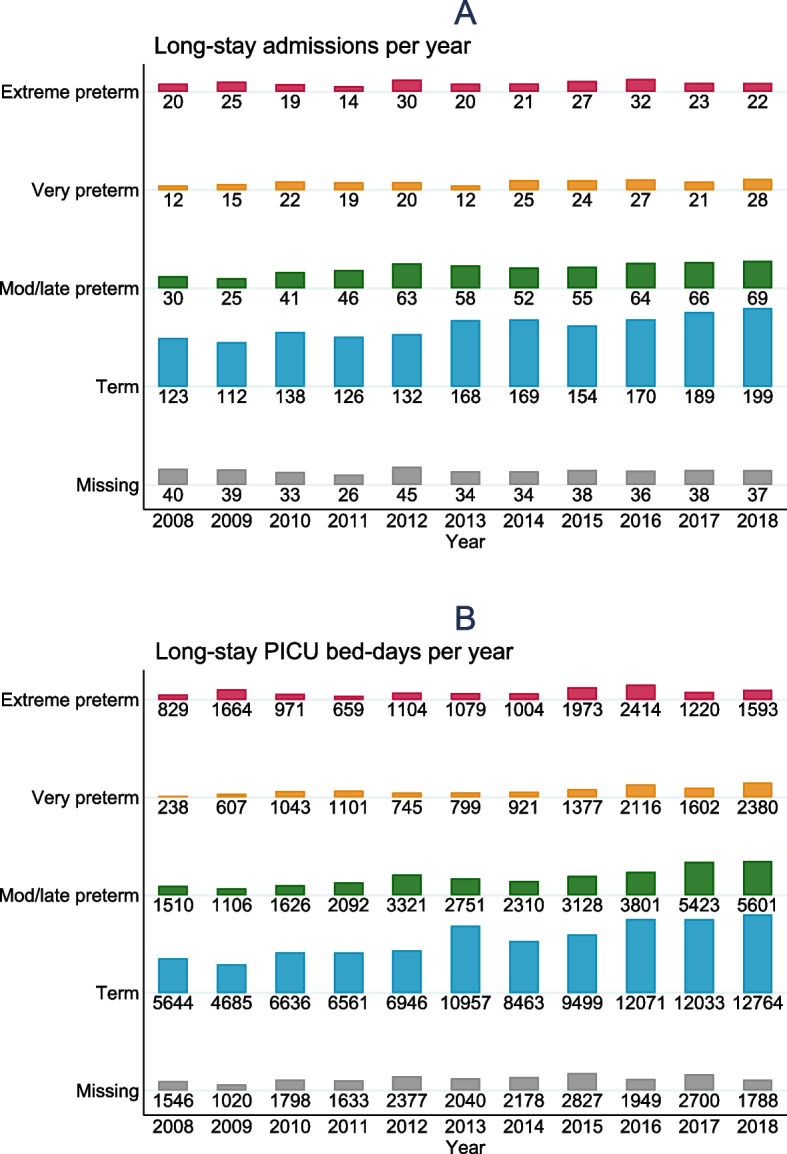
Annual number of long-stay PICU admissions (**A**) and bed days (**B**) for children aged <2 years in England 2008-2018, by gestational age at birth

Examining the number of long-stay admissions each year by gestational age at birth, there were statistically significant trends for very preterm (*p*=0.016), moderate preterm (*p*=0.001) and term-born (*p*<0.001) admissions showing increases over time (Fig 2A). However, this was not the case for the extreme preterm-born admissions (*p*=0.242).

Similarly, there were significant trends showing increases in the number of PICU bed days over time for the very preterm (*p*=0.002), moderate preterm (*p*<0.001) and term (*p*<0.001) children, but not for those born extremely preterm (*p*=0.087) (Fig 2B).

**Fig. 3:**
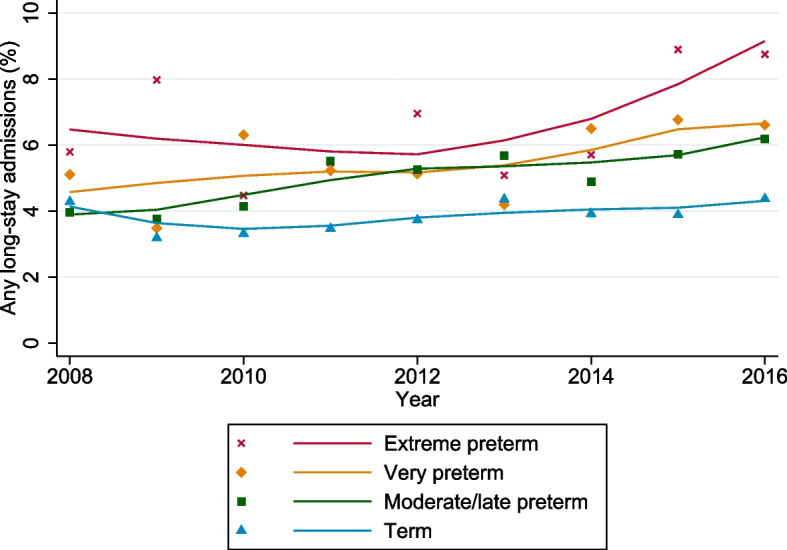
Percentage of children admitted to PICU who require a long-stay on any admission up to two years of age, comparison by gestational age at birth

During the analysis period, 6.4% of extremely preterm born children admitted to PICU were observed to have one or more long-stays in PICU, compared with 5.5%, 5.2% and 4.0% respectively for very preterm, moderate/late preterm and term respectively. Moreover, following smoothing of year-to-year variation, there appeared to be a progressive year-on-year increase in the percentage of long-stay admissions observed for the extremely preterm, very preterm and moderately preterm born children requiring PICU (Fig 3), although this only reached statistical significance in the moderate/late preterm group (*p*=0.009).

Because of the high level of missing data for gestation within the dataset, we performed sensitivity analyses for trends in the percentage of children with PICU long-stays. Firstly, we assumed that admissions with missing gestational age at birth were born at term (see Additional file, Supplementary Figure 2), which showed similar results to initial analysis. Furthermore, we repeated our analysis including only units with high data completeness (<20% missing) for gestational age (11 PICUs, 44,831 children, 66.3%) which again confirmed our results, although the difference between extreme preterm and other preterm groups was less marked (see Additional file, Supplementary Figure 3) (extreme preterm 6.0%, very preterm 5.5%, moderate/late preterm 5.1%, term 3.8%).

### Details of PICU care for long-stay admissions

There were 634,663 days of PICU care delivered to children aged under two years, of which 178,223 (28.1%) were for long-stay admissions.

For children born at term, 28.3% of care days were delivered to children with long-stay (Table 2). This proportion was greater (31.3%) for those born preterm. We had data on the care delivery, interventions and therapies for 97.7% of total PICU bed days. PICU care delivered for long-stay patients differed from that for shorter admissions: there was a smaller proportion of bed days in which children received endotracheal tube ventilation for long-stay admissions, but greater proportions for tracheostomy ventilation and non-invasive ventilation. Among preterm-born children there was a greater use of tracheostomy ventilation, up to 41.8% of care days for extreme preterm-born long-stay children, compared to 2.6% for term-born standard stay children.

**Table 2:** Comparison between PICU bed days for admissions ≥28 days (long-stay) and <28 days (standard stay)

	**Study population**	**Term**^**a**^		**Preterm**^**a**^					
				**All preterm**			**Extreme preterm (<28 weeks)**		
		**Standard stay**	**Long-stay**	**Standard stay**	**Long-stay**	***p* (long- stay: preterm vs term)**	**Standard stay**	**Long-stay**	***p* (long- stay: extreme preterm vs term)**
**Bed-days, n (%)**	634,663 (100)	244,060 (71.7)	96,259 (28.3)	131,698 (68.7)	60,108 (31.3)	<0.001	30,449 (67.7)	14,510 (32.3)	<0.001
**Bed-days receiving endotracheal tube ventilation, n (%)**	337,555 (53.2)	140,327 (57.5)	43,880 (45.6)	76,119 (57.8)	23,612 (39.3)	<0.001	16,986 (55.8)	4,354 (30.0)	<0.001
**Bed-days receiving tracheostomy ventilation, n (%)**	62,761 (9.9)	6,263 (2.6)	27,270 (28.3)	6,094 (4.6)	19,407 (32.3)	<0.001	1,870 (6.1)	6,071 (41.8)	<0.001
**Bed-days receiving non-invasive ventilation, n (%)**	81,426 (12.8)	24,886 (10.2)	14,409 (15.0)	16,188 (12.3)	10,848 (18.1)	<0.001	4,155 (13.7)	2,610 (18.0)	<0.001
**Bed-days receiving ECMO, n (%)**	9,796 (1.4)	4,239 (1.7)	2,194 (2.3)	1,159 (0.9)	587 (1.0)	<0.001	211 (0.7)	69 (0.5)	<0.001
***Missing data for interventions***	*14,630 (2.3)*	*7,845 (3.2)*	*243 (0.3)*	*4,170 (3.2)*	*167 (0.3)*	-	*1,285 (4.2)*	*52 (0.4)*	-

*ECMO* Extra-corporeal membrane oxygenation therapy

^a^102,538 bed days (16.2%) had missing data for gestation at birth, therefore not shown in comparison by gestation

Among term-born children, a greater proportion of PICU care delivery involved extracorporeal membrane oxygenation therapy (ECMO) for long-stay children compared to standard stay (2.3% of care days vs 1.7%). However, among the preterm-born children these proportions were lower (0.5% to 1.0%) and were not higher among the long-stay population.

## Discussion

This large population-based study of PICU admissions in children aged under two years of age in England demonstrated an increasing trend of long-stay admissions and long-stay bed utilisation. Preterm birth was associated with long-stay after adjustment for other admission characteristics, and there were significant increases in long-stay admissions among preterm-born children over the study period. Children with long-stay PICU admissions had a significantly higher mortality, and morbidity as demonstrated by readmissions to PICU and need for tracheostomy ventilation. While preterm-born children with long-stays did not have a higher mortality than term-born children with long-stays, children born extremely preterm had the greatest use of tracheostomy ventilation and readmissions to PICU. These results have significant implications for PICU capacity, service delivery and planning for future service provision.

Even accounting for missing data for gestational age, it is clear that preterm-born children were over-represented within the long-stay population; only 8% of births in the UK are preterm and <0.5% of live births are under 28 weeks [11], compared to 33.5% of children with long-stay admissions as born preterm, and 8.4% born extremely preterm. The number of long-stay admissions to PICU by children born preterm is increasing, most notably for those born very preterm and moderate/late preterm, who may still be affected by complications of prematurity particularly if combined with other conditions such as congenital heart disease [12]. However, it should be noted that between 2008 and 2018 total annual PICU admissions in England increased from 8,354 to 8,866, which may partially account for these increases. Among preterm-born children admitted to PICU, a greater percentage experienced PICU long-stays compared to term-born children, and this appeared to be increasing.

Our data confirms a higher mortality rate for children with long-stay in PICU compared to those without long stay as found previously [4], although this was not fully reflected in the admission PIM2 scores for predicted mortality among long-stay children, most likely as the presence of medical complexity does not influence the PIM2 calculation. The observed mortality was not disproportionately higher in preterm-born children, this may be due to effect of selecting those preterm babies who did not die in neonatal care, who go on to be admitted to PICU. Whilst the preterm-born population was similar in terms of mortality, they had higher levels of healthcare need. This included more transfers from neonatal care to PICU, and more PICU readmissions, further highlighting the high levels of morbidity, and the prolonged periods of intensive care these children experience. The first admission to PICU frequently occurred early, before 44 weeks PMA, and as a high proportion of children with long-stays are cared for by both neonatal and PICU clinicians, greater collaboration between the two groups of healthcare professionals could improve the care delivered to these children.

### The impact of preterm birth on long-stay admissions and medical complexity

The ongoing effects of preterm birth beyond the neonatal period have been well described, for example ongoing abnormalities in lung function [13] and increased rates of asthma and airways disease [14] in childhood following BPD. Children born preterm have increased healthcare utilisation compared to those born at term [8], and are generally over-represented within PICUs [15, 16]. Previous studies have suggested that outcomes within PICUs for children born preterm are worse compared to those born at term including: increased duration of PICU stay [16], increased mechanical ventilation [17], and increased mortality [16]. In addition, children born preterm with congenital heart disease requiring surgery have greater mortality and morbidity than those born at term, which impacts on their post-operative care on PICU [12].

The increase in long-stay admissions that we observed is consistent with two previous single centre studies from the United Kingdom and Spain [4, 5]. Previous single-centre studies have also identified that between 11% and 18% of children with long-stays in PICU are born preterm, so over-represented compared to the preterm birth rate of 7% to 8% [18], and moreover this proportion appears to be increasing [5, 15]. However, these studies are limited in their sample sizes and generalisability. Focussing on children aged under two years, we found an even greater proportion of children with long-stays were born preterm (33.5%) and our data adds further evidence that children born preterm are at risk of adverse outcomes extending beyond the perinatal period.

Previous studies have shown children with medical complexity form an increasing proportion of the PICU population [19, 20], with higher mortality than the general PICU population and a higher proportion of repeat PICU admissions [20]. Moreover, the population of children receiving long-term ventilation has increased [21, 22], and has high requirements for PICU services [23]. There is evidence that children with complex respiratory needs are contributing to PICU long-stays, for example a high prevalence of tracheostomies in the long-stay population [4]. Preterm birth has also been linked to medical complexity: between 28% and 40% of babies discharged home dependent on technology are born preterm [24, 25], and preterm-born children make up between 18% and 77% of those undergoing tracheostomy formation [26, 27].

In our study, among the long-stay population non-invasive and tracheostomy ventilation was more frequent, which may represent children with long-term ventilation, and this proportion increased with prematurity. This may contribute to the over-representation of preterm-born children within the long-stay population. This association may be due to airways disease (such as subglottic stenosis) or BPD following preterm birth – respiratory disease was the most common primary diagnosis for long-stay preterm-born children. An alternative explanation for the increasing long-term ventilation population could be neurodevelopmental disability, however the prevalence of major neurodevelopmental impairment and cerebral palsy following extreme preterm birth has decreased in recent decades [28]. Further research is required to better understand the nature of multiple comorbidities for this group of children, and the contribution to PICU admissions.

### Policy implications of this study

Our data examines admissions which took place following the release of the British Association of Perinatal Medicine (BAPM) Framework for Management of Babies Born Extremely Preterm in 2008 [29], which advised that some babies born at 23 weeks may benefit from neonatal intensive care but was clear in advising against survival-focussed care for babies born at 22 gestational weeks. The subsequent updated framework in 2019, released after our study period, recommended that babies born at 22 weeks may be offered survival-focussed care [6]. Consequently, the population of children born most preterm, and therefore likely to have some of the greatest medical complexity, may increase further with potential impact on PICU services – highlighting the importance of ongoing evaluation of changes in PICU care. On the other hand, in our study there were greater absolute increases in the number of very preterm and moderate/late preterm long-stay admissions, and so the impact of births at 22 to 24 weeks may be limited in the context of total PICU service provision.

Since the peak in 2012, the number of live births in England has fallen [11]. Despite this, the number of PICU admissions and PICU bed days per year increased until 2020, which saw a fall in PICU activity following the SARS-CoV-2 pandemic restrictions [3], which is unlikely to be sustained. As we have demonstrated, a considerable and increasing proportion of PICU activity is caring for children with long-stays. Preterm-born children form a large part of this demand, and there is possible additional unmet need considering those babies requiring critical care currently remaining in neonatal care beyond 44 weeks post-menstrual age, who should potentially be cared for within PICUs [30], and require effective managed transition of their care.

As the rate of change in the nature of the paediatric population dependent on critical care is outstripping the rate of change in service development, the burgeoning cohort of children with medical complexity in paediatric critical care is putting pressure on the capacity of PICU and the ability to provide adequate staffing [31]. New models of care should be considered for managing patients in this cohort who require long-term critical care, including delivery of care outside of PICUs. The recent Paediatric Critical Care National Review from NHS England identified this problem and suggested that development of level 2 (high dependency) capacity outside the PICU should be prioritised and adequately resourced [31]. The review also recommended the formation of Operational Delivery Networks (ODNs) able to facilitate collaborative working between PICUs within tertiary centres and level two units in non-specialist hospitals. The Paediatric Critical Care GIRFT (Getting It Right First Time) Programme National Specialty Report published in April 2022 identified similar challenges in care delivery for children with long-term respiratory support outside of PICU, and also described the ongoing challenges in developing effective ODNs across England and Wales [30]. Our study provides further evidence that the recommendations of these reports should be implemented, to allow children to receive critical care in the appropriate setting, and deliver a sustainable service within the NHS.

### Strengths and limitations

A strength of this work is that we have used a national population-level dataset including eleven years of admissions to address an important area of paediatric research, and examine an expanding group of children with considerable morbidity. As all admissions aged under two in England were included, our study is more generalisable than previous single-centre studies. In this study we focussed on children aged under two years, who make up the majority of the PICU population, it should be noted that PICANet does not request data for gestation at birth of children aged two and over [3].

Whilst the level of missing data was not inconsequential, we undertook a robust sensitivity analysis to provide confidence in our findings. When examining trends in annual long-stay admissions by gestation, the number of admissions with missing data for gestation was static, however as a proportion of overall admissions those with missing data for gestation decreased over the period, which may complicate interpretation of trends. Absolute numbers of annual long-stay admissions by extreme preterm-born children were relatively low, which may have resulted in type II error when testing statistical significance of trends over time.

It should be noted that for the outcome of readmission to PICU, due to left-censoring of children whose first admission was before the study period (i.e. in 2006 or 2007), we may have under-counted readmissions for those children who were readmitted to PICU in 2008 and 2009. However, the proportion of long-stay children with PICU readmissions was considerable despite this, and was similar to the readmission rate described previously in children with medical complexity [20].

In this study we were not able to examine critical care occurring within neonatal intensive care units; to overcome this limitation we plan to link data from the National Neonatal Research Database and PICANet to better understand these patient journeys. Moreover, our data may be influenced by geographic variation in care models, for example some regions have access to tertiary paediatric services within neonatal care whereas others must transfer babies to a PICU [30].

Understanding the epidemiology of this population of children is important, however future research should also investigate the ethical and medico-legal developments relevant to children with medical complexity, and the efficacy of interventions to prevent or manage chronic critical illness.

## Conclusion

In conclusion, we have shown that long-stay admissions to PICU of babies and young children are increasing within England, and that children born preterm and those receiving long-term respiratory support contribute to this complex population. Furthermore, the long-stay population experiences substantially higher mortality rates compared to other PICU admissions, and commonly experiences multiple readmissions. Despite decreases in the birth rate within the United Kingdom [11], the demand for paediatric critical care is increasing [3]. This may be driven by increasing numbers of children born extremely preterm who survive neonatal care [6] with ongoing morbidity requiring long-stay PICU admissions. While only a minority of children discharged from neonatal care may require PICU, within PICU they comprise a considerable proportion of admissions. Further studies are required to better understand the effects of increasing survival following extreme preterm birth, and further planning at a national level is required to provide optimal critical care services for this group of children, including developing delivery of level 2 critical care and effective Operational Delivery Networks.

### Supplementary Information

**Additional file 1: Supplementary Figure 1.** Flow diagram of PICU admissions included for analysis. **Supplementary Figure 2.** Percentage of children admitted to PICU who require a long-stay on any admission up to two years of age, comparison by gestational age at birth, assuming that missing values were birth at term. **Supplementary Figure 3.** Percentage of children admitted to PICU who require a long-stay on any admission up to two years of age, comparison by gestational age at birth, including only data from units with <20% missing data for gestation. **Supplementary Table 1.** Logistic regression analyses for any long-stay ≥28 days, from first PICU admission characteristics, comparing results of primary model (Model 1) and sensitivity analyses (Models 2 and 3).

## Data Availability

PICANet collects data on the PICU referrals, transfers, and PICU admissions of all children in the UK and Ireland. Data is available by request for use in audit and research. Data on PICU admissions within England can be accessed via a data request to PICANet and the Healthcare Quality Improvement Partnership. See www.picanet.org.uk/data-collection/data-requests for further information. The PICANet dataset manual is available from: https://www.picanet.org.uk/data-collection/data-manuals-and-guidance/ For queries regarding data used in this study please contact the corresponding first author Dr Tim van Hasselt (t.vanhasselt@nhs.net), or email PICANet for Data and Information Requests (picanetdataaccess@leeds.ac.uk).

## Abbreviations

PICU: Paediatric intensive care unit
BPD: Bronchopulmonary dysplasia
ECMO: Extracorporeal Membrane Oxygenation therapy
UK: United Kingdom
NHS: National Health Service
ODN: Operational Delivery Network
PIM 2: Paediatric Index of Mortality 2 score
PMA: Postmenstrual age
aOR: Adjusted odds ratio
95%CI: 95% Confidence Intervals
