# Supplementary material for: Impact of prematurity on long-stay paediatric intensive care unit admissions in England 2008-2018
Source: BMC Pediatr. 2023 Aug 24;23:421. doi: 10.1186/s12887-023-04254-0 (PMC10463455; doi:10.1186/s12887-023-04254-0)
Supplement: Supplementary file 1 — Additional file 1: Supplementary Figure 1. Flow diagram of PICU admissions included for analysis. Supplementary Figure 2. Percentage of children admitted to PICU who require a long-stay on any admission up to two years of age, comparison by gestational age at birth, assuming that missing values were birth at term. Supplementary Figure 3. Percentage of children admitted to PICU who require a long-stay on any admission up to two years of age, comparison by gestational age at birth, including only data from units with <20% missing data for gestation. Supplementary Table 1. Logistic regression analyses for any long-stay ≥28 days, from first PICU admission characteristics, comparing results of primary model (Model 1) and sensitivity analyses (Models 2 and 3). [file 12887_2023_4254_MOESM1_ESM.docx]

**Additional files**

**Supplementary Figure 1 – Flow diagram of PICU admissions included for analysis**

174,164 admissions to PICUs in England aged 0-18 years, 2008-2018

Excluded 75,093 admissions
 aged >2 years

99,071 admissions to PICUs in England aged <2 years, 2008-2018

Excluded 14 admissions with missing length of stay data

Gestational age <22+0 (n=6) or ≥43+0 (n=29) changed to missing – children not excluded

99,057 admissions to PICUs in England aged <2 years, 2008-2018, with length of stay data (67,615 individual children)

Study population for analysis:
99,057 PICU admissions,
67,615 individual children

**Supplementary Figure 2 - Percentage of children admitted to PICU who require a long-stay on any admission up to two years of age, comparison by gestational age at birth, assuming that missing values were birth at term**


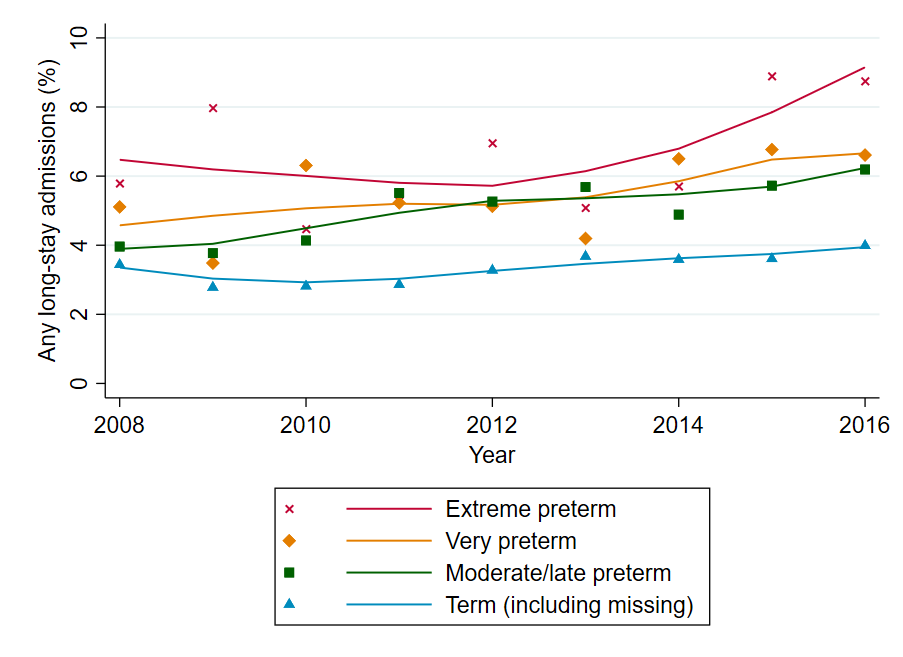


**Supplementary Figure 3 - Percentage of children admitted to PICU who require a long-stay on any admission up to two years of age, comparison by gestational age at birth, including only data from units with <20% missing data for gestation**


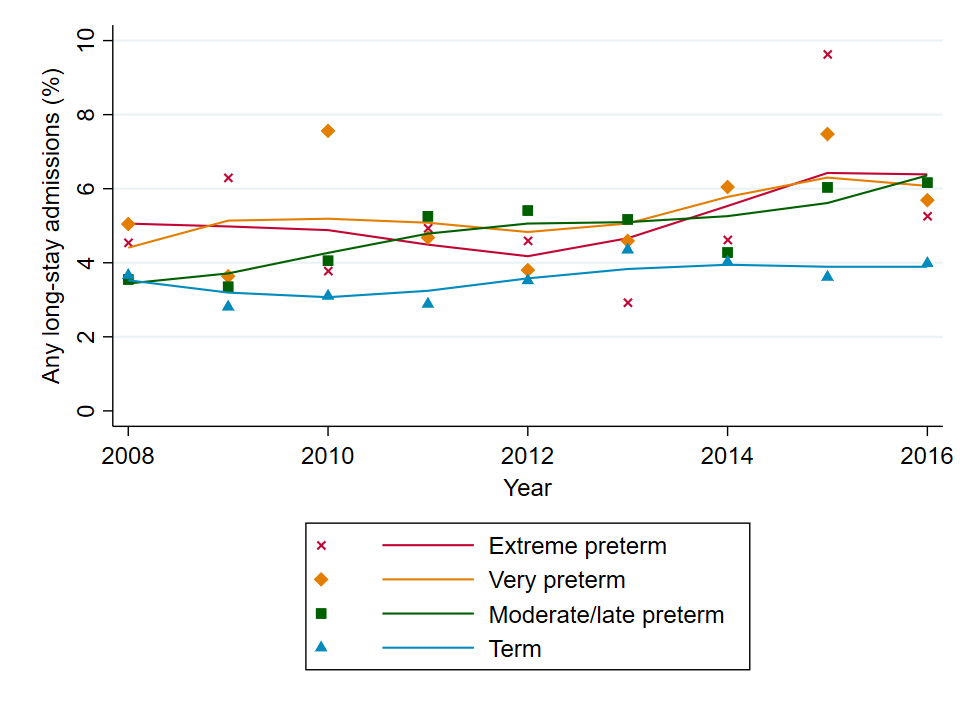


**Supplementary Table 1 – Logistic regression analyses for any long-stay ≥28 days, from first PICU admission characteristics, comparing results of primary model (Model 1) and sensitivity analyses (Models 2 and 3).**

|  |  | **Model 1: Complete case analysis** | | **Model 2: Replace missing for term** | | **Model 3: PICUs with high data-completeness only** | |
| --- | --- | --- | --- | --- | --- | --- | --- |
|  | **N** | **51,646** | | **66,536** | | **39,357** | |
|  | **c-statistic** | **0.685** | | **0.703** | | **0.6814** | |
|  | **Brier Score** | **0.0813** | | **0.0813** | | **0.0909** | |
|  |  | **aOR (95% CI)** | ***p*** | **aOR (95% CI)** | ***p*** | **aOR (95% CI)** | ***p*** |
| **Gestation at birth (ref: term)** | **Term** | Reference | - | Reference | - | Reference | - |
|  | **Moderate/late preterm** | 1.276 (1.142 to 1.426) | <0.001 | 1.380 (1.237 to 1.539) | <0.001 | 1.281 (1.124 to 1.460) | <0.001 |
|  | **Very preterm** | 1.348 (1.146 to 1.585) | <0.001 | 1.453 (1.237 to 1.707) | <0.001 | 1.351 (1.116 to 1.636) | 0.002 |
|  | **Extreme preterm** | 1.303 (1.116 to 1.521) | 0.001 | 1.385 (1.188 to 1.615) | <0.001 | 1.199 (0.994 to 1.445) | 0.058 |
| **Age (corrected if <37/40)** | **(months)** | 0.981 (0.972 to 0.991) | <0.001 | 0.976 (0.967 to 0.984) | <0.001 | 0.976 (0.965 to 0.988) | <0.001 |
| **Sex** | **Male** | Reference | - | Reference | - | Reference | - |
|  | **Female** | 1.091 (1.002 to 1.188) | 0.045 | 1.117 (1.032 to 1.209) | 0.006 | 1.115 (1.010 to 1.232) | 0.031 |
| **Primary diagnosis group** | **Respiratory** | Reference | - | Reference | - | Reference | - |
|  | **Neurological** | 0.625 (0.509 to 0.767) | <0.001 | 0.602 (0.498 to 0.728) | <0.001 | 0.568 (0.444 to 0.725) | <0.001 |
|  | **Cardiovascular** | 1.143 (1.028 to 1.271) | 0.014 | 1.188 (1.076 to 1.312) | 0.001 | 0.999 (0.882 to 1.132) | 0.991 |
|  | **Infection** | 0.531 (0.419 to 0.672) | <0.001 | 0.521 (0.418 to 0.649) | <0.001 | 0.512 (0.385 to 0.680) | <0.001 |
|  | **Gastrointestinal** | 0.543 (0.450 to 0.654) | <0.001 | 0.562 (0.471 to 0.671) | <0.001 | 0.525 (0.426 to 0.648) | <0.001 |
|  | **Other** | 0.903 (0.782 to 1.043) | 0.167 | 0.868 (0.756 to 0.996) | 0.044 | 0.790 (0.666 to 0.937) | 0.007 |
| **PIM2 (%, grouped)** | **<1%** | Reference | - | Reference | - | Reference | - |
|  | **1% to <5%** | 1.263 (1.105 to 1.443) | 0.001 | 1.374 (1.211 to 1.560) | <0.001 | 1.284 (1.088 to 1.516) | 0.003 |
|  | **5% to <15%** | 2.145 (1.863 to 2.470) | <0.001 | 2.537 (2.221 to 2.899) | <0.001 | 2.250 (1.891 to 2.677) | <0.001 |
|  | **15% to <30%** | 3.025 (2.533 to 3.613) | <0.001 | 3.649 (3.091 to 4.308) | <0.001 | 3.201 (2.590 to 3.957) | <0.001 |
|  | **>30%** | 3.069 (2.525 to 3.731) | <0.001 | 3.465 (2.883 to 4.164) | <0.001 | 3.469 (2.756 to 4.366) | <0.001 |
| **Previous neonatal or intensive care during hospitalisation** | **None** | Reference | - | Reference | - | Reference | - |
|  | **Mixed/adult ICU** | 1.139 (0.836 to 1.551) | 0.410 | 1.371 (1.057 to 1.779) | 0.018 | 1.179 (0.806 to 1.724) | 0.397 |
|  | **PICU** | 2.705 (2.211 to 3.310) | <0.001 | 2.596 (2.157 to 3.124) | <0.001 | 2.805 (2.231 to 3.528) | <0.001 |
|  | **Neonatal unit** | 1.939 (1.751 to 2.148) | <0.001 | 1.925 (1.748 to 2.120) | <0.001 | 1.829 (1.620 to 2.064) | <0.001 |
